# Comment on “Bidirectional Mendelian randomization and single-cell sequencing reveal T cell-mediated causal links between COPD and lung adenocarcinoma”

**DOI:** 10.1097/JS9.0000000000003223

**Published:** 2025-08-20

**Authors:** Yuanli You, Mingxing Yang, Wen Dong

**Affiliations:** Department of Respiratory Medicine, Hainan Cancer Hospital, Haikou City, Hainan Province China

*Dear Editor,*

We read with great interest the article titled “Bidirectional Mendelian randomization and single-cell sequencing reveal T cell-mediated causal links between COPD and lung adenocarcinoma^[1]^.” This well-conducted study offers valuable insights into the immune mechanisms linking COPD and lung cancer, and we commend the authors for their contribution to the field. However, we would like to address a few concerns that may impact the completeness and applicability of the findings. This correspondence was developed following the TITAN Guidelines 2025^[2]^ for the declaration and use of artificial intelligence (AI) in scholarly publishing . No AI tools were employed in the conception, design, analysis, interpretation, or writing of this work.

Firstly, the study primarily focuses on single-cell RNA sequencing (scRNA-seq) data from **peripheral blood** samples, which, while valuable, may not fully capture the immune environment specific to the lung, where the disease processes are most active. COPD and lung adenocarcinoma involve complex immune interactions in the lung tissue, and previous research has shown that the immune cell composition in the lung differs significantly from that in peripheral blood^[3]^. Therefore, we believe the inclusion of **lung tissue samples**, such as those obtained via bronchoscopy or biopsy, would provide a more comprehensive view of the disease mechanisms. The authors might consider adding this as a limitation in the manuscript and recommend that future studies incorporate both peripheral blood and lung tissue samples to enhance the robustness of the conclusions.

Secondly, while the study employs strict quality control measures in scRNA-seq data analysis, **doublet removal**, a critical step in ensuring the accuracy of single-cell data, is not explicitly mentioned. Doublets, where two cells are captured as one, can introduce substantial noise and bias in downstream analyses^[4,5]^. Given the significant impact of doublets on single-cell RNA-seq results, we recommend that the authors clarify whether doublet removal was applied.

We believe that addressing these concerns will not only improve the transparency and completeness of the study but also enhance its clinical applicability, particularly in the context of lung disease research.”

## Data Availability

The letter does not have any data.

## Research registration unique identifying number (UIN)

Not registered for the type of the study.
